# Deep Learning-Based Culture-Free Bacteria Detection in Urine Using Large-Volume Microscopy

**DOI:** 10.3390/bios14020089

**Published:** 2024-02-05

**Authors:** Rafael Iriya, Brandyn Braswell, Manni Mo, Fenni Zhang, Shelley E. Haydel, Shaopeng Wang

**Affiliations:** 1 Biodesign Center for Biosensors and Bioelectronics, Arizona State University, Tempe, AZ 85287, USA; ririya@asu.edu (R.I.); bbraswe1@asu.edu (B.B.); mannimo@asu.edu (M.M.); fenni.zhang@asu.edu (F.Z.); shelley.haydel@asu.edu (S.E.H.); 2 School of Electrical and Computer Engineering, Tempe, AZ 85287, USA; 3 School of Molecular Sciences, Arizona State University, Tempe, AZ 85287, USA; 4 School of Life Sciences, Arizona State University, Tempe, AZ 85287, USA; 5 School of Biological and Health Systems Engineering, Arizona State University, Tempe, AZ 85287, USA

**Keywords:** bacteria detection, UTI diagnostics, deep learning, CNN, light scattering microscopy, LVM

## Abstract

Bacterial infections, increasingly resistant to common antibiotics, pose a global health challenge. Traditional diagnostics often depend on slow cell culturing, leading to empirical treatments that accelerate antibiotic resistance. We present a novel large-volume microscopy (LVM) system for rapid, point-of-care bacterial detection. This system, using low magnification (1–2×), visualizes sufficient sample volumes, eliminating the need for culture-based enrichment. Employing deep neural networks, our model demonstrates superior accuracy in detecting uropathogenic *Escherichia coli* compared to traditional machine learning methods. Future endeavors will focus on enriching our datasets with mixed samples and a broader spectrum of uropathogens, aiming to extend the applicability of our model to clinical samples.

## 1. Introduction

Urinary tract infections (UTIs) are among the most prevalent infections globally, affecting various parts of the urinary system, including the bladder, kidneys, ureters, and urethra [1,2]. Often, UTIs have high recurrence rates, specifically in women, necessitating periodic checkups and underlining the importance of effective management [3]. *Escherichia coli* (*E. coli*) is the primary causative agent of UTIs, though a range of other organisms, including bacteria like *Klebsiella pneumoniae* and *Proteus mirabilis* as well as fungi, also contribute to these infections [2,3,4]. The typical treatment regimen for UTIs involves the use of antibiotics, which, while effective, also highlights the importance of appropriate usage to mitigate broader health concerns such as antimicrobial resistance (AMR) [5,6,7].

Furthermore, UTIs represent a significant medical and economic challenge. In the United States, approximately 50% of women are projected to experience a UTI by the age of 35, and men account for 20% of all UTI cases [3,8]. This results in an annual healthcare cost of approximately USD 3.5 billion in the US alone [2]. The considerable impact of these infections underscores the urgent need for efficient and accurate diagnostic and treatment methods. This urgency is amplified by global concerns regarding AMR, highlighting the importance of prudent antibiotic usage alongside effective UTI management [6,7,9,10].

Diagnosing UTIs typically involves evaluating urinary symptoms and confirming diagnoses with urine cultures, which detect the presence of pyuria and bacteriuria [11]. Despite being the standard diagnostic method, urine cultures have their drawbacks, such as varying thresholds for detection and a time-consuming nature, often requiring at least two days to provide results [12,13]. To complement this method, urine dipstick tests are used widely due to their rapidity and low cost. These tests detect indicators of infection, such as nitrite and leukocyte esterase, but are limited by a sensitivity range of 65% to 82%. They can yield inaccurate results, particularly in cases involving pathogens that do not produce nitrite or when certain antibiotics are used [14,15,16].

Given the economic impact of UTIs and the significant proportion of antibiotic prescriptions they account for, coupled with the limitations of current diagnostic methods, there is a pressing need for more immediate, convenient, and economical diagnostic approaches [12,13]. Recent advancements in point-of-care testing (POCT) for UTIs have shown promise in improving the speed and accuracy of bacteriuria detection, ranging from sophisticated biosensors to portable analyzers [17,18,19,20]. However, these methods still face challenges such as limited sensitivity to certain bacteria and dependency on user expertise.

In response to these challenges, our research introduces a novel method that combines our developed large-volume microscopy (LVM) [21,22] with deep learning algorithms. LVM provides a rapid, culture-free technique for detecting a broad range of bacteria in urinary samples, thus addressing the limitations of current POCTs. The integration of deep learning [22,23,24,25,26,27] enhances the ability to analyze large datasets with high precision, overcoming the issues related to species-specific detection and traditional diagnostic delays. This innovative approach not only promises to enhance the accuracy and speed of UTI diagnostics but also aligns with the imperative to manage AMR more effectively. By offering a potentially more versatile and comprehensive tool for UTI diagnosis, our method positions itself as a potential solution to the global healthcare challenges posed by UTIs and AMR.

## 2. Materials and Methods

### 2.1. Imaging Device Setup and Analysis

#### 2.1.1. LVM

**Figure 1:**
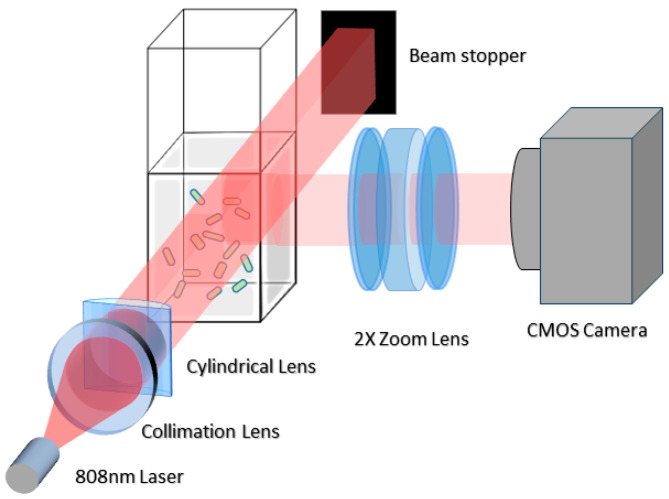
Optical configuration of LVM setup. This diagram illustrates the key optical elements in our LVM system: a near-infrared multimode diode laser is collimated through a collimation lens and forms a light slab via a cylindrical lens to illuminate sample in a cuvette. The exit beam is blocked via a beam stopper. A CMOS camera equipped with a variable zoom lens (set at 2× zoom) captures side scattering images of the sample.

The LVM system setup, illustrated in Figure 1 (modified from [21]), consists of a cuvette holding the sample, which is then illuminated by a light slab from a laser. This illumination generates a series of side scattering images, recorded at 2× magnification using a CMOS camera. The resulting grayscale images, characterized by a wide view and high depth of field, displayed samples of *E. coli*, urine particles, and polystyrene beads (see Supplementary Material Figure S1 for particle examples). All particles were visible as bright blobs with a brighter center and a Gaussian decay extending outward. These particles exhibited intermittent blinking due to micromotion, oscillating through peaks and valleys over time (Supplemental Figure S2). Additionally, the laser-induced thermal drift caused the particles to move along curved paths. These movement patterns, essential for particle detection, are depicted in Supplemental Figure S3.

In raw images captured by the CMOS camera, both beads and *E. coli* displayed similar gray value intensity distributions prior to background subtraction. The background in these images exhibited a consistent intensity range of 900–1100 gray values. In contrast, the intensity distribution for both beads and bacteria was markedly higher, ranging between 8000 and 30,000 gray values. This significant difference in intensity values was further corroborated through a point spread function (PSF) analysis of individual particles. Notably, the intensity of these particles varied, which can be attributed to the influence of Brownian motion on their behavior and the consequent scattering of light.

#### 2.1.2. Algorithm

**Figure 2:**
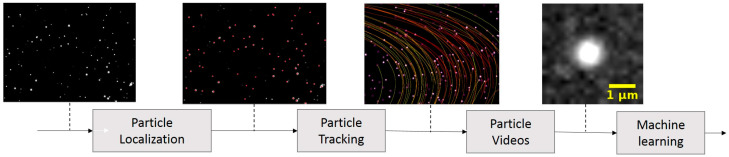
Block diagram of the developed algorithm illustrates the sequence of transformations applied to the original LVM video. Background subtraction is followed by the application of ImageJ (version 1.52g) TrackMate (version 4.0.1) for particle identification. Once particles are located, they are tracked through subsequent frames using a custom-developed Kalman filtering algorithm. The tracked particles are then isolated into segments of 12 × 12 pixels for intensity analysis. The varying colors of the tracks represent differences in track lengths.

The algorithm developed for our study comprised several steps, as depicted in Figure 2. The process began with the LVM video undergoing a background subtraction process, where the minimum value for each pixel was computed over intervals of 600 frames. The LVM video contained sequential images of different particles to include polystyrene beads, bacteria, and urine particles. In this paper, we classify urine particles as small amounts of tissue, protein aggregation, blood and skin cells, and amorphous crystals typically found in healthy patients’ urine. The following step involved a particle localization algorithm that identified the centroids of these particles. Subsequently, these centroids were tracked across video frames using our tracking algorithm. This tracking process resulted in the creation of new videos featuring single particles, as illustrated in Figure 2. These single-particle videos were then utilized in the machine learning algorithm for two primary purposes: training the model and classifying the particles. To improve the specificity of our method in detecting *E. coli*, we designed binary models that have the potential to differentiate *E. coli* from both polystyrene beads and urine particles.

#### 2.1.3. Particle Localization

The particle localization process in our study utilized ImageJ [28] with the Trackmate plugin [29]. The particle centroids were found using Laplacian of Gaussian (LoG) detector, which is effective for detecting edges across varying image scales and degrees of focus [30]. We employed Trackmate’s LoG detector in ImageJ, setting estimated object diameter = 5 and a quality threshold = 10. Estimated object diameter was chosen based on the average diameter of particles in both conditions. Quality threshold was then chosen to remove noise after both conditions were analyzed with an object diameter of 5. Additionally, we incorporated sub-pixel localization from Trackmate’s selection options. This step was crucial for enhancing the accuracy of identifying edges for particle localization. It is important to note that while we utilized the blob detection feature of Trackmate, the tracking of particles was conducted with our own algorithm, details of which are provided in the subsequent section.

#### 2.1.4. Tracking

The tracking component of our system was based on a modified Kalman filter algorithm our lab implemented previously [27]. Our adaptation included two major modifications tailored to the characteristics of LVM particles. Firstly, since LVM particles do not exhibit multiple contrasts, all aspects of the original implementation related to contrast changes were omitted. Secondly, to account for the predominant drift in particle motion caused by thermal induction from the laser, we incorporated drift velocities into the algorithm for more precise position estimates when searching for the particle in the subsequent frame.

The tracking algorithm operates with a search radius (Sr) of 6 pixels and accommodates missing frames (MT) up to 50 and shared locations (CT) up to 100 frames. Drift estimation was conducted by dividing each frame image into a grid of 10 × 10 regions. Within each region (R), drift (d) was calculated using a moving average d_avg_:

d(t,R) = αd_avg_ (t,R) + (1 − α)d(t − 1,R) (1)

Here, t represents time, and α, set at 0.2 and ranging from 0 to 1, is a constant chosen to allow for a gradual update of the drift values. This gradual update is essential for accurately tracking the particle’s trajectory between frames, considering the non-linear nature of its motion. d_avg_(t,R) is determined by averaging the difference between posteriori position estimates over two adjacent frames for all particles in the same region. Each particle was assigned to the region whose center is nearest to its current frame location. An example of drift vectors in a frame is shown in Supplemental Figure S4. Furthermore, the posteriori velocity vector v = [v_x_,v_y_]^T^ for each particle (P) was updated using another moving average formula:

v(t,P) = βd(t,R) + (1 − β)v(t − 1,P) (2)

β, also ranging from 0 to 1 and chosen as 0.8, plays a significant role in accounting for most of the particle’s motion, which is predominantly non-linear. This choice of β value ensures that the drift comprehensively represents the particle’s movement.

#### 2.1.5. Single-Particle Video Generation

After obtaining particle tracks, we filtered out unreliable tracks by a minimum duration (Min_dur_ = 650 frames/16.25 s) and by a full width at half maximum (FWHM) threshold (T_FWHM_ = 5). T_FWHM_ of 5 represents the signal-to-noise ratio of the particle to background after the background subtraction algorithm has been applied. The minimum duration avoids having particles generate two or more tracks due to faulty tracking (the LVM duration is 1200 frames/30 s), and the FWHM eliminates out-of-focus particles, removing particles with short or long width/short peak. Single-particle videos were generated by zooming in on the original LVM videos at the particle centroid on each frame, then cropping a region of 12 × 12 pixels. The size of the region was chosen to minimize the number of background pixels, while keeping the entire particle visible.

#### 2.1.6. Machine Learning vs. Deep Learning Comparison

In our study, we conducted a comparative analysis between our deep learning approach and a traditional machine learning approach. The traditional approach, involving handcrafted features, feature selection, and support vector machines (SVMs), extracted a set of 93 features focusing on spatial and temporal aspects for each single-particle video. This approach used the sequential forward selection (SFS) algorithm, a greedy search method aimed at minimizing the number of selected features while maintaining model efficacy [31]. For the classification task, SVMs were chosen for their proficiency in identifying optimal separation hyperplanes in binary classifications [32], utilizing radial basis function (RBF) kernels.

**Figure 3:**
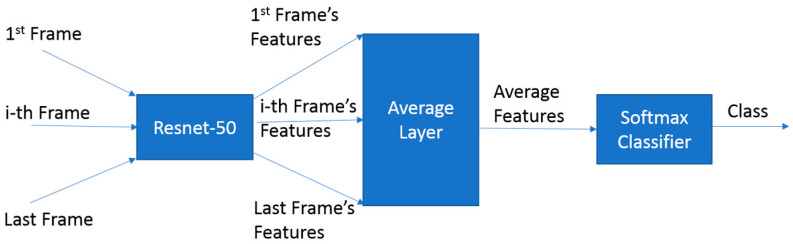
Structure of CNNFA architecture.

In contrast, our deep learning approach utilized advanced neural network architectures for automatic feature extraction. Initially, we employed a CNN-LSTM network [33], combining a convolutional neural network (CNN) for spatial feature extraction and long short-term memory (LSTM) neural network for temporal features, enhanced with an attention mechanism [34] to encourage the model to utilize features from various points in the time series. However, due to overfitting issues with this model, we adopted an alternative approach inspired by the Youtube-8M benchmark [35]. This second model, CNN with feature averages (CNNFA), averages CNN features from all frames before the classification layer as shown in Figure 3. This method, mirroring the Youtube-8M benchmark’s approach to video-based classification, was more in tune with our dataset, particularly in handling its complexities and avoiding the overfitting challenges faced by the attention-based model.

For both models, a ResNet-50 [36] was chosen as the base CNN network due to its standard application in handling large datasets. ResNet architectures, known for their efficiency, maintain a low error rate even with increased network depth, making them ideal for our complex spatial and temporal feature extraction tasks. In addition to selecting ResNet-50, several key implementation strategies were pivotal in training our robust deep learning models:

- Mean subtraction: implementing mean subtraction, where the mean was calculated as a scalar for each single-particle video by averaging over all pixels and frames, improved accuracy by up to 5%;
- The use of L2-norm regularization and a dropout rate of 0.5, a common practice in deep learning, significantly reduced overfitting. This dropout rate is particularly effective in preventing co-adaptation of neurons during training, thereby enhancing the convergence of validation cross entropy loss;
- Dynamic range adjustment: to improve loss convergence, dynamic range adjustment was performed, capping the maximum intensity value at 1000 and normalizing all values between 0 and 1. We replaced zero values with the smallest non-zero value in each video, which is a critical adjustment considering the original particle pixel range of 0–65,535. This step was necessary to prevent instabilities in the model caused by extreme intensity variations. The highest non-zero value considered as zero is part of this normalization process, ensuring a stable and efficient analysis framework;
- Learning rates: small learning rates, ranging from 10^−7^ to 10^−5^, were employed to ensure proper convergence of validation loss;
- Video length: the length of videos was critical to provide sufficient particle temporal information for training robust models;
- Class balancing and randomization: to ensure class balance, the number of training samples for each class was equalized in each epoch. Furthermore, samples were randomized in each epoch to maximize the utilization of the dataset for training.

#### 2.1.7. Datasets

Our study employed distinct datasets for two classification challenges: discrimination between *E. coli* and beads (Dataset 0) and an independent analysis of *E. coli* and urine particles (Datasets 1, 2, and 3). The design of these datasets was driven by the need to encompass a broad range of variability, testing our model’s robustness against factors like sample-to-sample irregularities and variations in the system’s state. Each dataset was structured by day, sample, and replicate, with a replicate being a cuvette containing only one type of particle to ensure sample homogeneity. This arrangement allowed for a comprehensive analysis under varied conditions, such as changes in camera configuration or environmental factors like thermal drift caused by the laser. By capturing data across different days and conditions, we aimed to simulate real-world scenarios where these variables might impact the model’s performance.

For each sample, along with its replicates, we produced individual LVM videos. Hundreds of single-particle videos were generated from these videos, each uniformly labeled with the particle type from the original sample. This separation of particle types within each sample was critical for our analysis. It ensured a consistent ground truth and eliminated the possibility of mixing particle types, allowing us to rigorously test the model’s capability to classify under varied and challenging conditions.

Dataset 0: This dataset focused on discriminating between *E. coli* and beads. It comprised data collected over four different days. On each day, we prepared one sample with four replicates for both *E. coli* and beads. Typically, one LVM video per sample and replicate generated about 100–300 individual 12 × 12-pixel videos for each particle tracked, leading to approximately 500–1500 videos per sample. For the phases of training and validation, we utilized data from the first three days, resulting in a dataset of 1500–4500 videos. Data from the fourth day, containing another set of 500–1500 videos, were reserved for testing.

Datasets 1–3: These datasets were dedicated to a separate analysis of *E. coli* and urine particles.

- Dataset 1: This dataset included data from a single day, with one *E. coli* sample and one urine particle sample, each accompanied by four replicates. The first replicate from each category was used for validation, contributing 100–300 videos. For testing, we used a separate day’s data, also consisting of one *E. coli* and one urine particle sample, along with their replicates, providing an additional 500–1500 videos. The training phase encompassed approximately 400–1200 videos;
- Dataset 2: This dataset featured data from one day, but with four distinct samples (and their respective four replicates) for each particle type. The first sample set was allocated for validation, yielding 500–1500 videos, while the training dataset included 1500–4500 videos. For testing, we used data from a different day, mirroring the sample and replicate structure, resulting in another 500–1500 videos;
- Dataset 3: This dataset encompassed data spanning six days. The first sample (and its replicates) from each day was designated for validation, amounting to a total of 3000–9000 videos. The training phase incorporated data from the remaining samples, tallying up to 12,000–36,000 videos. Testing was conducted with data from a different day, producing an additional 2000–6000 videos.

Accuracy was defined as the ratio of correctly identified particles (both true positives and true negatives) to the total number of cases (true positives, false positives, true negatives, and false negatives). Our model training involved datasets containing a mix of labeled videos, each distinctly featuring either *E. coli*, beads, or urine particles. This approach enabled us to evaluate the model’s ability to accurately classify the particle type in each 12 × 12-pixel video, thereby assessing its proficiency in independently discriminating between the particle types under study.

### 2.2. Materials and Reagents

#### 2.2.1. LVM Setup

The LVM setup was composed of a 1 W, 808 nm multimode diode laser (L808P1000MM, Thorlabs Inc., Newton, NJ, USA) with a collimation lens and a cylindrical lens (Thorlabs Inc., Newton, NJ, USA) to focus the laser beam into a light slab through the sample. Side scattering images were recorded by a CMOS camera (Flea3 FL3-U3-13S2M, Point Gray Research Inc., Richmond, BC, Canada) for 30 s at 800 × 600-pixel resolution, 40 fps, and 2× magnification through a variable zoom lens (NAVITAR 12×, Navitar, New York, NY, USA) placed at a 90° angle to the laser light beam. The image volume (1.81 μL) was determined by the size of the light slab that illuminated the sample and by the viewing size and focal depth of the optics (1.85 mm × 1.4 mm × 0.7 mm).

#### 2.2.2. Sample Preparation

Uropathogenic *E. coli* ATCC25922 was purchased from American Type Culture Collection (ATCC) and stored at −80 °C in 5% glycerol. *E. coli* cells were cultured overnight for approximately 16 h at 35 °C in lysogeny broth (LB) and then for additional ~2 h at 16 °C to an OD_600_ of ~0.7, corresponding to 5–6 × 10^8^ cfu/mL. *E. coli* cells were then collected by centrifugation and resuspended in phosphate-buffered saline to appropriate cell concentrations. Polystyrene beads (1 μm) were purchased from Bangs Laboratories, Inc (Fishers, IN, USA) and suspended in phosphate-buffered saline. Pooled healthy human urine (Lot number: BRH1311635) purchased from BioIVT (Westbury, NY, USA) was filtered using 5 μm syringe filters and then diluted 1:10. Samples were diluted until fewer than 1000 particles were counted with the particle localization algorithm, resulting in a concentration of approximately 10^5^ particles/mL.

#### 2.2.3. Data Processing

Data were processed using custom scripts for the ImageJ (version 1.52g) (TrackMate (version 4.0.1)), MATLAB, and Python programs, developed using the methods discussed above.

#### 2.2.4. Deep Learning

Models were trained using the Keras framework with the Tensorflow backend [37]. Images were resized to 20 × 20 pixels and models were trained for 40 epochs, using the Adam optimizer, 38 with a batch size of 32 [38]. The learning rate was chosen depending on the classification problem (10^−5^–10^−7^), as some datasets required a smaller learning rate to converge properly.

## 3. Results and Discussion

### 3.1. Accuracy Performance

Table 1 showcases the CNNFA method’s performance across the datasets.

Dataset 0, which focuses on discriminating between *E. coli* and beads, demonstrated a high training accuracy, suggesting effective differentiation based on size and shape variations. However, the dip in test accuracy, in contrast to training and validation, indicates potential issues in generalizing across different daily samples.

Dataset 1, addressing the classification of *E. coli* and urine particles, showed good training and validation accuracy but experienced a significant drop in test accuracy. This decline suggests the model’s struggle in generalizing across different samples of *E. coli* and urine particles, possibly due to variations in physical characteristics like size, shape, and light scattering properties.

Dataset 2 presented lower validation scores, underlining the challenges posed by physical variability in samples. This dataset’s results emphasize the impact of sample diversity on the model’s ability to accurately classify particle types.

These patterns across the datasets highlight that sample-to-sample variability, particularly in terms of physical properties and behavior under Brownian motion, is a significant challenge in developing robust classification models. The learning curves for all datasets are included in Supplemental Figure S5.

### 3.2. Method Comparison

We compared the performance of SVM, CNN-LSTM, and CNNFA for Dataset 0 and Dataset 3 in Table 2 and Table 3, respectively. Dataset 0 revealed SVM’s weaker performance in training and validation for bead classification (53% and 52%) compared to CNNFA and CNN-LSTM (93% and 94% validation accuracies). This stresses SVM’s poor generalization, evident from its high test accuracy (93%).

In Dataset 3, which introduced more complex data variability, CNN-LSTM displayed weaker test performance, particularly for urine particles (21%), while CNNFA maintained higher validation accuracy (75% for *E. coli*, 82% for urine particles) compared to SVM (74% and 57%). Despite CNNFA’s performance dip, this dataset’s complexity, with its broader range of samples and separate testing data, offers valuable insights. It highlights CNNFA’s strengths in handling varied data, indicating potential areas for further optimization and application in diverse, real-world scenarios.

### 3.3. Effect of Dynamic Range Adjustment

**Figure 4:**
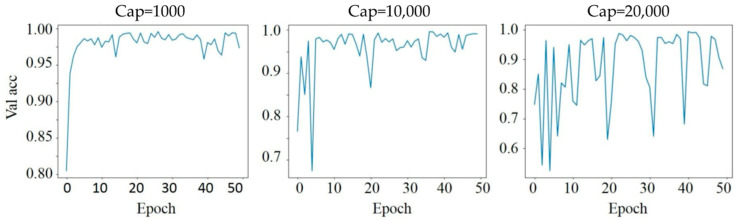
Effect of dynamic range adjustment with different intensity caps.

Dynamic range adjustment impacts model stability, as reflected in Figure 4. By capping the maximum intensity at different thresholds (1000, 10,000, and 20,000), we observed varying effects on validation accuracy stability over epochs. A cap of 1000 maintained more consistent validation accuracy, whereas higher caps, such as 10,000 and 20,000, led to greater fluctuations. This can be attributed to the way light intensity values are normalized, impacting the model’s ability to discern patterns within the data. The cap of 1000 likely offers a more focused range, reducing the influence of extreme pixel intensity values that could destabilize the learning process. The dataset, from a single day and sample of the *E. coli* and beads, provided a controlled environment to assess these effects.

### 3.4. Effect of Video Length

**Figure 5:**
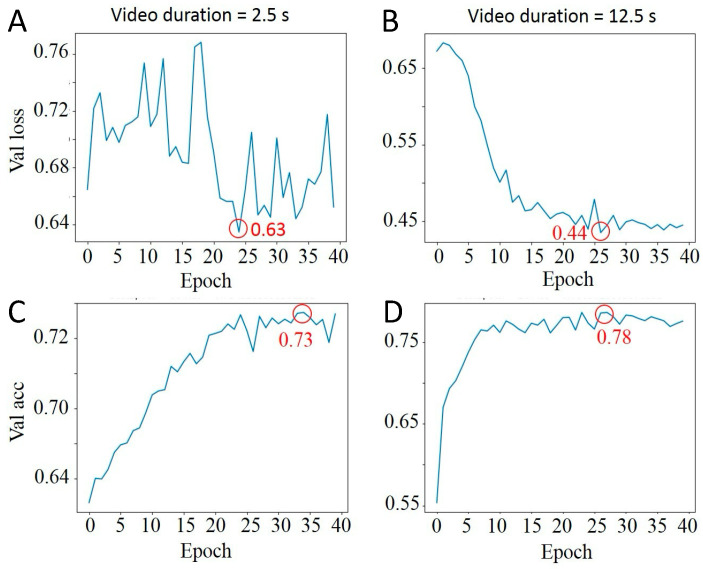
Comparison of different video lengths on CNNFA learning curves. (**A**) Lost function of 2.5 s video; (**B**) Lost function of 12.5 s video; (**C**) Validation accuracy of 2.5 s video; (**D**) Validation accuracy of 12.5 s video. Minimum lost function values and maximum validation accuracy values are circled in red.

We assessed how the length of the video—short (2.5 s) versus long (12.5 s)—affects the learning curves of our CNNFA model, as demonstrated in Figure 5. Longer videos led to slightly better maximum validation accuracy, with only a 3% difference. However, the loss function for short videos was less stable, exhibiting significant oscillation across epochs. This instability suggests that longer video durations provide more reliable data for training, likely due to the increased temporal information allowing for more robust feature extraction by the CNNFA model. To obtain 12.5 s videos, the first 500 frames from the track were selected and then time-averaged every 5 frames to reduce the amount of training data.

### 3.5. Effect of Setup Configuration

We also evaluated the effect of modifying certain parameters on the setup by training models in one setup configuration and testing using different configurations. The following parameters were assessed:

- Laser position in X, Y, Z planes;
- Camera position in X, Y, Z planes;
- Lights turned off;
- Digital controlled field of view (center, top left, and bottom right).

We evaluated the effects of various setup parameters on the classification model’s performance by training with one setup configuration and testing with various changes to the setup. The default setup configuration, referred to in our experiments, was the origin point with coordinates (0,0,0) for both laser and camera positions, which we consider our control condition. The default setup also maintained the lights on within the testing room. Changes in laser and camera positions are denoted in millimeters (mm) along the respective axes.

Assessing the impact of these modifications on model accuracy revealed that the camera’s *X*-axis position and the digital field of view critically influenced the classification results due to their effect on scattered light collection as well as particle density and tracking. Other setup variables, such as laser alignment and ambient lighting conditions, contributed by altering the angle of incidence and background noise caused by a secondary light. These findings, documented in Table 4, accentuate the necessity of maintaining a consistent experimental setup to ensure the robustness and reproducibility of our classification models.

## 4. Conclusions

Our study presents significant advancements in bacterial detection through a customized LVM system, enhanced by a novel deep learning algorithm and our application of CNNFA. These developments demonstrate potential in diverse classification tasks and mark a significant step forward in the realm of point-of-care diagnostics.

The results of our study, particularly the accuracy performance across datasets and comparisons with various machine learning models, shows the potential of our approach. While the high accuracy in differentiating *E. coli* and beads in Dataset 0 and the improved generalization in Dataset 3 with varied training data are encouraging, they also highlight the importance of expanding our research beyond *E. coli*. Although *E. coli* is the most common causative agent of UTIs, the incidence of several other uropathogens cannot be overlooked. Future studies will aim to include a broader range of bacterial species to ensure our method’s applicability across a more comprehensive spectrum of urinary tract infections.

We also recognize the challenges posed by the dataset’s size and diversity. The variability in accuracy scores suggests that a more extensive and diverse dataset is crucial for enhancing the algorithm’s robustness and reliability. Expanding the dataset will allow for more effective adaptation to complex clinical scenarios.

Looking ahead, the potential application of our LVM system in practical point-of-care diagnostics is a long-term goal that requires further development and validation. Addressing the nuances of dataset size and diversity, the effects of setup configuration, and balancing accuracy with generalization are critical for our future work. By expanding our focus to include various uropathogens and enriching our datasets, we aim to refine our system to meet the challenges of real-world diagnostic applications. This will contribute to more efficient and accurate detection of urinary tract infections, thereby enhancing patient care and management.

## Figures and Tables

**Table 1:** Results for all datasets using CNNFA.

Dataset	*E. coli* Accuracy (%)			Bead/Urine Particle Accuracy (%)		
	Train	Val	Test	Train	Val	Test
0	97	93	95	94	89	78
1	100	93	56	98	92	85
2	89	95	84	62	61	40

**Table 2:** Method comparison for Dataset 0.

Method	*E. coli* Accuracy (%)			Bead Accuracy (%)		
	Train	Val	Test	Train	Val	Test
SVM	52	53	93	96	90	75
CNN-LSTM	97	94	95	95	89	79
CNNFA	97	93	95	94	89	78

**Table 3:** Method comparison for Dataset 3.

Method	*E. coli* Accuracy (%)			Bead Accuracy (%)		
	Train	Val	Test	Train	Val	Test
SVM	78	74	79	62	57	50
CNN-LSTM	77	89	80	57	46	21
CNNFA	64	75	63	89	82	60

**Table 4:** Comparison of training and testing on different setup configurations.

Configuration	*E. coli* Accuracy (%)	Urine Particle Accuracy (%)
Default (training)	81	72
Laser Z (−10)	79	74
Laser Y (+20)	81	72
Laser Y (−10)	79	74
Laser X (+20)	81	72
Laser X (+20)	79	74
Cam Z (+10)	81	72
Cam Z (−10)	79	74
Cam Y (+20)	81	72
Cam Y (−10)	79	74
Cam X (+20)	81	72
Cam X (+10)	79	74
Cam X (−10)	81	72
Lights Off	81	72
Field of view (bottom right)	79	74
Field of view (top left)	79	74

## Data Availability

Due to the large size of the image dataset, the data are available from the corresponding author upon reasonable request.

## Supplementary Materials

The following supporting information can be downloaded at: https://www.mdpi.com/article/10.3390/bios14020089/s1, Figure S1: Visualization of (a) beads, (b) *E. coli*, and (c) urine particles using the LVM setup; Figure S2: Intensity plot of a bead particle revealing intermittent blinking; Figure S3: Bead tracks revealing thermal drift; Figure S4: Example of drift velocity vectors; Figure S5: Learning curves showing the progression of cross-entropy loss for the tested datasets; Table S1: Handcrafted features tested with SVM and SFS; Table S2: Dataset 0 (*E. coli* and beads); Table S3: Dataset 1 (*E. coli* and urine); Table S4: Dataset 2 (*E. coli* and urine); Table S5: Dataset 3 (*E. coli* and urine).
