# Supplementary material for: Deep Learning-Based Culture-Free Bacteria Detection in Urine Using Large-Volume Microscopy
Source: Biosensors (Basel). 2024 Feb 5;14(2):89. doi: 10.3390/bios14020089 (PMC10887190; doi:10.3390/bios14020089)
Supplement: Supplementary file 1 [file biosensors-14-00089-s001.zip › biosensors-2829258-supplementary.pdf]

Deep Learning-Based Culture-Free Bacteria Detection in Urine Using Large-Volume Microscopy

Rafael Iriya [1,2], Brandyn Braswell [1,3], Manni Mo [1,3], Fenni Zhang [1], Shelley E. Haydel [1,4] and Shaopeng Wang [1,5,*]

1 Biodesign Center for Biosensors and Bioelectronics, Arizona State University, Tempe, AZ 85287, USA; ririya@asu.edu (R.I.); bbraswe1@asu.edu (B.B.); mannimo@asu.edu (M.M.); fenni.zhang@asu.edu (F.Z.); shelley.haydel@asu.edu (S.E.H.)
2 School of Electrical and Computer Engineering, Tempe, AZ 85287, USA
3 School of Molecular Sciences, Arizona State University, Tempe, AZ 85287, USA
4 School of Life Sciences, Arizona State University, Tempe, AZ 85287, USA
5 School of Biological and Health Systems Engineering, Arizona State University, Tempe, AZ 85287, USA
* Correspondence: shaopeng.wang@asu.edu

Supplemental Figure S1

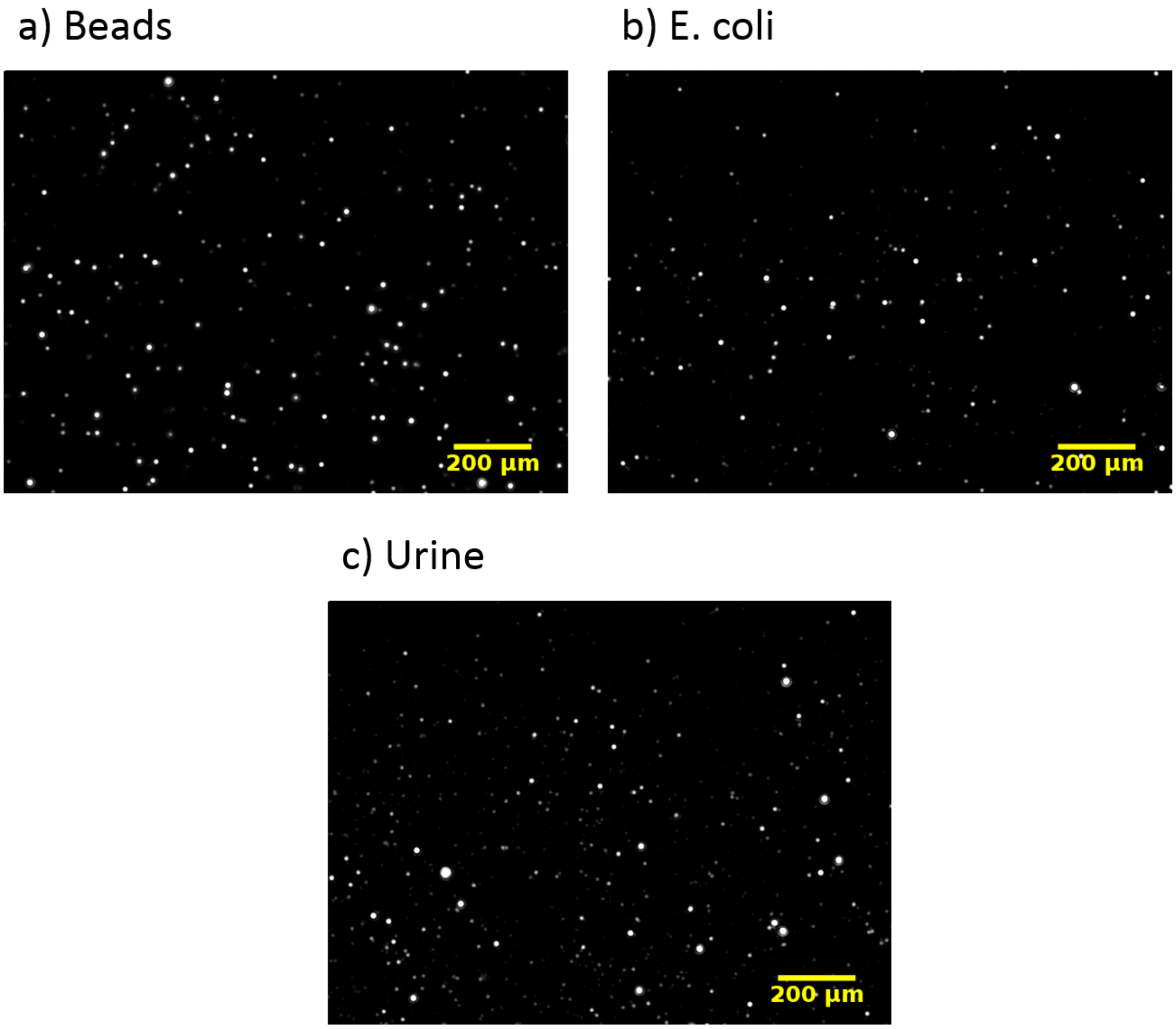

Figure S1. Visualization of a) beads, b) *E. coli*, and c) urine particles using the LVM setup.

Supplemental Figure S2

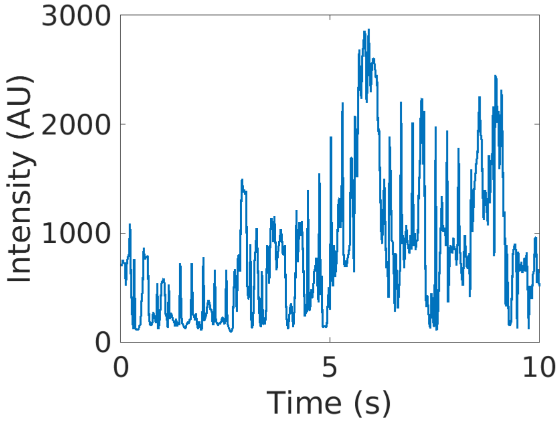

Figure S2. Intensity plot of a bead particle revealing its blinking nature.

Supplemental Figure S3

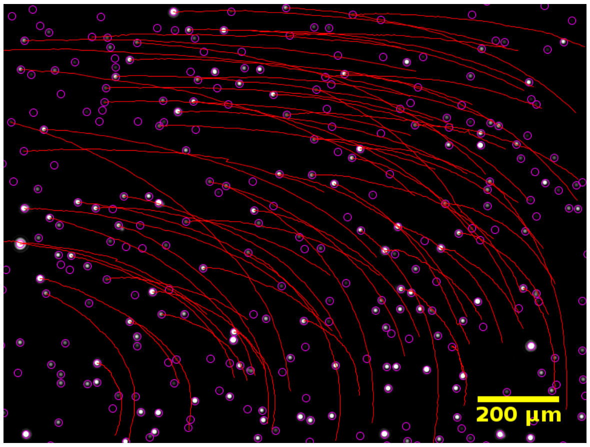

Figure S3. Bead tracks revealing the drift.

Supplemental Figure S4

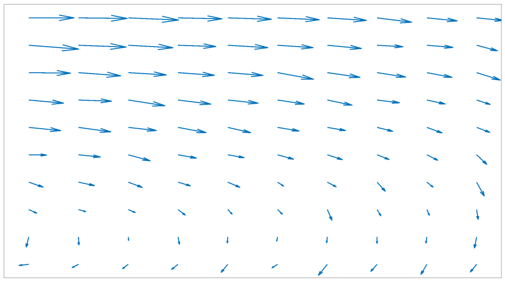

Figure S4. Example of drift velocity vectors.

Supplemental Figure S5

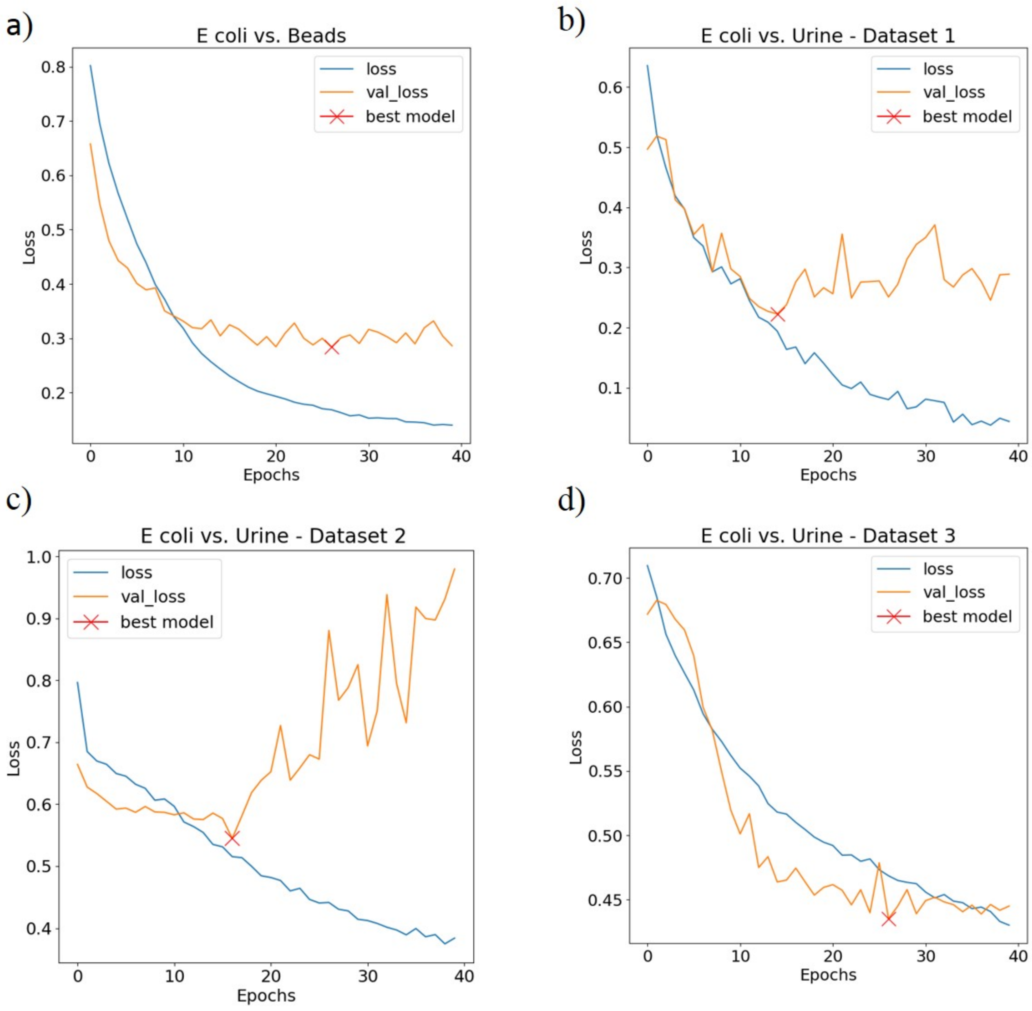

Figure S5. Learning curves showing the progression of the cross-entropy loss for the tested datasets.

Supplemental Table S1

MeanPercentMissingFrames	KurtStdIntensity	1stDerivKurtQuality
MeanPeakDist	KurtQuality	2ndDerivMeanCenterIntensity
MeanDetectionRate	1stDerivMeanCenterIntensity	2ndDerivMeanArea
MeanCenterIntensity	1stDerivMeanArea	2ndDerivMeanAvgIntensity
MeanArea	1stDerivMeanAvgIntensity	2ndDerivMeanStdIntensity
MeanAvgIntensity	1stDerivMeanStdIntensity	2ndDerivMeanQuality
MeanStdIntensity	1stDerivMeanQuality	2ndDerivStdCenterIntensity
MeanQuality	1stDerivStdCenterIntensity	2ndDerivStdArea
StdCenterIntensity	1stDerivStdArea	2ndDerivStdAvgIntensity
StdArea	1stDerivStdAvgIntensity	2ndDerivStdStdIntensity
StdAvgIntensity	1stDerivStdStdIntensity	2ndDerivStdQuality
StdStdIntensity	1stDerivStdQuality	2ndDerivPctl25CenterIntensity
StdQuality	1stDerivPctl25CenterIntensity	2ndDerivPctl25Area
Pctl25CenterIntensity	1stDerivPctl25Area	2ndDerivPctl25AvgIntensity
Pctl25Area	1stDerivPctl25AvgIntensity	2ndDerivPctl25StdIntensity
Pctl25AvgIntensity	1stDerivPctl25StdIntensity	2ndDerivPctl25Quality
Pctl25StdIntensity	1stDerivPctl25Quality	2ndDerivPctl75CenterIntensity
Pctl25Quality	1stDerivPctl75CenterIntensity	2ndDerivPctl75Area
Pctl75CenterIntensity	1stDerivPctl75Area	2ndDerivPctl75AvgIntensity
Pctl75Area	1stDerivPctl75AvgIntensity	2ndDerivPctl75StdIntensity
Pctl75AvgIntensity	1stDerivPctl75StdIntensity	2ndDerivPctl75Quality
Pctl75StdIntensity	1stDerivPctl75Quality	2ndDerivSkewCenterIntensity
Pctl75Quality	1stDerivSkewCenterIntensity	2ndDerivSkewArea
SkewCenterIntensity	1stDerivSkewArea	2ndDerivSkewAvgIntensity
SkewArea	1stDerivSkewAvgIntensity	2ndDerivSkewStdIntensity
SkewAvgIntensity	1stDerivSkewStdIntensity	2ndDerivSkewQuality
SkewStdIntensity	1stDerivSkewQuality	2ndDerivKurtCenterIntensity
SkewQuality	1stDerivKurtCenterIntensity	2ndDerivKurtArea
KurtCenterIntensity	1stDerivKurtArea	2ndDerivKurtAvgIntensity
KurtArea	1stDerivKurtAvgIntensity	2ndDerivKurtStdIntensity
KurtAvgIntensity	1stDerivKurtStdIntensity	2ndDerivKurtQuality

Table S1. Handcrafted features tested with SVM and SFS.

Supplemental Table S2

Day	1	2	3	4
Training		x	x	
Validation	x			
Test				x

Table S2. Dataset 0 (*E. coli* and beads). Cross validation was applied for the first 3 days, which were used once for validation.

Supplemental Table S3

Day	1							
Sample	1				2			
Replicate	1	2	3	4	1	2	3	4
Training		x	x	x				
Validation	x							
Test					x	x	x	x

Table S3. Dataset 1 (*E. coli* and urine).

Supplemental Table S4

Day	1	2	3				4				5				6				7			
Sample			1	2	3	4													1	2	3	4
Training				x	x	x																
Validation			x																			
Test																			x	x	x	x

Table S4. Dataset 2 (*E. coli* and urine).

Supplemental Table S5

Day	1	2	3				4				5				6				7			
Sample	1	1	1	2	3	4	1	2	3	4	1	2	3	4	1	2	3	4	1	2	3	4
Training	x	x		x	x	x		x	x	x		x	x	x		x	x	x				
Validation			x				x				x				x							
Test																			x	x	x	x

Table S5. Dataset 3 (*E. coli* and urine).
